# Optimizing fungal treatment of lignocellulosic agro‐industrial by‐products to enhance their nutritional value

**DOI:** 10.1002/fsn3.4131

**Published:** 2024-04-08

**Authors:** Mohamed Benaddou, Hassan Hajjaj, Aimad Allali, Tarik Moubchir, Hasna Nait M’Barek, Hiba‐Allah Nafidi, Yousef A. Bin Jardan, Fakhreldeen Dabiellil, Mohammed Bourhia, Mariyem Chakir, Mohammed Diouri

**Affiliations:** ^1^ Biotechnology and Bio‐Resource Development Laboratory (BioVar) Moulay Ismail University Zitoune Meknes Morocco; ^2^ Laboratory of Plant, Animal and Agro‐Industry Productions University of Ibn Tofail Kenitra Morocco; ^3^ Polyvalent Team in Research and Development, Department of Biology Faculté Polydisciplinaire Beni Mellal Beni‐Mellal Morocco; ^4^ Department of Food Science, Faculty of Agricultural and Food Sciences Laval University Quebec City Quebec Canada; ^5^ Department of Pharmaceutics, College of Pharmacy King Saud University Riyadh Saudi Arabia; ^6^ Department of Translation, Faculty of Arts University of Khartoum Khartoum Sudan; ^7^ Laboratory of Biotechnology and Natural Resources Valorization, Faculty of Sciences Ibn Zohr University Agadir Morocco

**Keywords:** cellulose, digestibility, fungal treatment, lignin, lignocellulosics, nutritional value, ruminant feed, sustainable agriculture

## Abstract

This study delves into the dynamic interaction between various fungal strains, substrates, and treatment durations to optimize the nutritional value of these by‐products. Six fungi, including *Penicillium chrysogenum*, *Fusarium* sp., *Fusarium oxysporum*, *Fusarium solani*, *Penicillium crustosum*, and *Cosmospora viridescens*, were evaluated across three substrates: wheat straw (WS), cedar sawdust (CW), and olive pomace (OP) over treatment periods of 4, 8, and 12 weeks. The study discerned profound impacts of these fungi across multiple parameters, including cellulose variation (C.var), lignin variation (L.var), and in vitro true digestibility variation (IVTD.var). Our results demonstrated that the various fungi had a significant effect on all parameters (*p* < .001). Noteworthy, *F. oxysporum* and *F. solani* emerged as exemplars, displaying notable lignin degradation, cellulose liberation, and IVTD enhancement. Importantly, *P. crustosum* demonstrated substantial cellulose degradation, exhibiting optimal efficacy in just 4 weeks for all substrates. Notably, *F.* sp. excelled, yielding favorable results when treating WS. *P. chrysogenum* achieved optimal outcomes with 8‐week treatment for WS. Both *Fusarium* sp. and *P. chrysogenum* exhibited slight cellulose release, with remarkable reduction of WS lignin compared to other substrates. Especially, WS and OP displayed superior digestibility enhancements relative to CW. It should be noted that the treatment duration further shaped these outcomes, as prolonged treatment (12 weeks) fostered greater benefits in lignin degradation and digestibility, albeit with concomitant cellulose degradation. These findings underscore the intricate balance between fungal strains, substrates, and treatment durations in optimizing the nutritional value of lignocellulosic agro‐industrial by‐products. The outcomes of this study lead to the enhancement in the overall value of by‐products, promoting sustainable livestock feed and advancing agricultural sustainability.

## Graphical abstract

Changes in NFC/NDF Ratio and Crude Protein Content of Lignocellulosic Substrates (OP: olive *pomace*, CW: Cedar wood sawdust, WS: wheat straw) Treated by various fungal strains (F. *solani*, F. *oxysporum*, F. sp, P. *chrysogenum*, P. *crustosum*, C. *viridescens*) Over a 12‐Week Duration. ^abc^difference in superscripts in the same substrate indicates significance at *p* < .05.

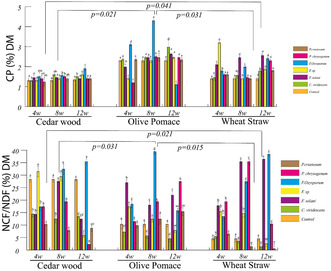

## 1 INTRODUCTION

The exponential growth of industrialization and globalization, coupled with population growth, has dramatically accelerated the expansion of agricultural and industrial operations worldwide (Wei et al., 2022; Yafetto et al., 2023). This has resulted in significant production of lignocellulosic biomass, which amounts to approximately 181.5 billion tons annually, of which 8.2 billion tons is used (Mujtaba et al., 2023). Morocco is characterized by its vibrant agro‐industrial landscape, which contributes significantly to its gross domestic product (GDP), with agriculture accounting for nearly 13%. Morocco is a notable producer of various agricultural by‐products, including industrial (such as olive, tomato, wasted potatoes (Chauhan et al., 2023), and wood by‐products) and agricultural (such as wheat straw) wastes amounting to around 4 million tons per year. According to the Ministry of Energy Transition and Sustainable Development, Department of Energy Transition (2023), the technical energy potential is about 13.4 million MWh (megawatt hour) per year. In parallel with Morocco's contributions, China plays a pivotal role as a colossal producer, contributing 126 million metric tons of wheat straw and accounting for 25% of the world's cotton production. The United States leads in the production of agricultural residues, contributing an impressive 80% of the global total, along with a substantial 25% of the world's sugarcane bagasse. In addition, Europe plays a significant role in the production of oat straw, accounting for a remarkable 64% of the world's total (Velvizhi et al., 2023). Unfortunately, a significant portion of these agro‐industrial by‐products receive inadequate posttreatment and are often incinerated or disposed of in makeshift landfills. This situation raises significant environmental and sustainability concerns.

The cell wall fraction of lignocellulosic biomass, which includes carbohydrates such as cellulose and hemicellulose (Tufail et al., 2021) and pectin, and the nonstructural or cell content fraction, which contains carbohydrates such as starch, sugars (water‐soluble carbohydrates; WSC), organic acids (OA), fructans, lipids, and proteins, have significant potential for sustainable ruminant production (Sufyan et al., 2024). Neutral detergent fiber (NDF) can be partially degraded to volatile fatty acids by ruminal microorganisms (fungi, bacteria, etc.) to provide energy for the organisms. Additionally, adjusting the ratio of nonfiber carbohydrates to neutral detergent fiber (NFC/NDF) can influence ruminal fermentation and microbial type in ruminants (Ma et al., 2022). Lignocellulosic feeds provide an alternative to conventional feedstuffs such as cereals and oilseeds, demonstrating their promise to promote environmentally friendly and economically viable practices (Chai et al., 2022; Cheng & Whang, 2022). This alternative is particularly relevant in regions facing challenges due to prolonged drought, such as Morocco (Castellani et al., 2017). However, studies have revealed that the utilization of these agro‐industrial by‐products in ruminant feed is suboptimal, primarily due to their low nutritional value (Bentil, 2021; Rouches et al., 2019; Sajid et al., 2022). This low nutritional value is attributed to factors such as the presence of lignin and the low accessibility of cellulose (Diouri & Wiedmeier, 2000; Guo et al., 2022; Sajid et al., 2022) along with the low crude protein (CP) and nonfiber carbohydrates (NFC) (Li, Duan, et al., 2023; Li, Gu, et al., 2023; Li, Liu, et al., 2023; Villalba et al., 2021). Additionally, the presence of inhibitors, such as weak acids (e.g., acetic acid), furans (e.g., furfural, hydroxymethylfurfural (HMF)), and phenolic compounds (Dasthban et al., 2009; Singh & Jana, 2023; Zha et al., 2014), can further contribute to the reduced nutritional quality of these biomass materials.

In response to this nutritional challenge, researchers have explored various methods to enhance the nutritional value of lignocellulosic biomass, with a particular focus on biological approaches using ligninolytic fungi. The biological approach offers significant advantages, such as environmental friendliness and economic affordability, over chemical and physical methods (Pantet al., 2022). Ligninolytic fungi, such as *Ganoderma lucidum*, *Lentinula edodes*, *Pleurotus eryngii*, *Pleurotus ostreatus*, *Trichoderma reesei*, *Mucor indicus* (Iyyappan et al., 2023), as well as *Penicillium* and *Fusarium* (Benatti & Polizeli, 2023), have shown promise in enhancing the digestibility of lignocellulosic substrates. However, despite this potential, there is a notable gap in the literature regarding universally accepted criteria for assessing the effectiveness of these fungi in improving the nutritional value of lignocellulosic biomass. In particular, these studies have not clearly defined generally accepted criteria for assessing the efficacy of the fungi used, or if such criteria exist, they have been applied in a limited manner. For instance, achieving high digestibility from a particular biomass after treatment with a specific fungus is undoubtedly a positive outcome. However, it is not sufficient to confirm the overall effectiveness of the fungus in biomass treatment. The potential of fungi lies in their ability to enhance nutritional value by improving digestibility, decreasing lignin content, reducing cellulose crystallinity, and maintaining or improving the different nutrient contents (e.g., CP, cellulose, and NFC) in lignocellulosic biomass. To determine true efficacy, it is imperative to conduct trials on different lignocellulosic materials, taking into account critical factors, such as the initial biomass components (including cellulose, lignin, NFC, and CP) and treatment duration. This comprehensive approach ensures a more accurate assessment of the versatility and applicability of the fungal treatment to different feedstock sources. While basidiomycetes have demonstrated considerable potential, it's essential to expand the scope of the investigation to include ascomycete fungi as well (Grace Barrios‐Gutiérrez et al., 2023). Exploring a broader range of fungal species can lead to a more holistic understanding of the potential applications of ligninolytic fungi in biomass treatment. Such inclusivity can uncover novel fungal candidates that may offer unique advantages or synergistic effects when applied to different lignocellulosic materials. The primary objective of this study is to establish a comprehensive set of criteria for the effective selection of fungi in the treatment of agro‐industrial by‐products, with the overarching goal of enhancing their nutritional value. To achieve this objective, we conducted a thorough comparison and evaluation of six different fungal strains: *Fusarium solani*, *Fusarium oxysporum*, *F*. sp., *Penicillium chrysogenum*, *Cosmospora viridescens*, and *Penicillium crustosum*. This evaluation encompassed three different types of lignocellulosic materials, namely wheat straw, olive pomace, and cedar sawdust. Furthermore, the study spanned three treatment durations of 4, 8, and 12 weeks.

## 2 MATERIALS AND METHODS

### 2.1 Collecting and preparing lignocellulosic substrates

Based on the cellulose crystallinity index (CI) and the chemical composition, three different types of agro‐industrial by‐products were selected for the study: wheat straw (WS) (CI = 34%), cedar sawdust wood (CW) (CI = 54%), and olive pomace (OP) (CI = 41.2%). Wheat straw was obtained from fields in El Hajeb region of Morocco, while OP was obtained from the Olea Food Company in Meknes, Morocco, and CW was obtained from the Ifrane National Park in Morocco. CW and WS were ground to a particle size of 0.5–1.5 cm, while OP particles were 2–5 mm long. This variation in substrate size was chosen to accurately represent their natural origin in the field. The substrates were then soaked in water at room temperature for 3 days to ensure complete water penetration. The excess water was then drained off, and the substrates were autoclaved at 121°C for 20 min (van Kuijk et al., 2015).

A quantity of substrate, containing approximately 30 g of dry matter, was weighed and placed in 250 mL glass bottles closed with cotton to allow contamination‐free air exchange. The bottles were then autoclaved at 121°C for 20 min and were kept at room temperature (25 ± 3°C) until use. A total of 72 bottles were prepared for the combinations of three substrates, six fungi, and four treatment times. All 72 combinations were replicated in three time periods.

### 2.2 Spawn preparation for different fungi

The fungi used were selected based on their utilized carbon source. Two fungi, *C. viridescens*, and *P. crustosum*, were selected from a culture medium with only cellulose (C) as a carbon source. Two other fungi, *F. oxysporum* and, *F*. sp., were selected from a culture medium with only lignin (L) as a carbon source. Finally, *F. solani* and *P. chrysogenum* were selected from a culture medium containing both C and L as carbon sources. The fungi were identified in the laboratory and stored in glycerol at −20°C (Nait M'Barek et al., 2019).

Initial culture of the selected fungi was carried out in Czapek medium: 3 g NaNO_3_, 1 g K_2_HPO_4_, 0.5 g MgSO_4_·7H2O, 0.5 g KCl, 0.01 g FeSO_4_, 30 g sucrose, and distilled water at a fixed volume of 1000 mL (Li, Duan, et al., 2023; Li, Gu, et al., 2023; Li, Liu, et al., 2023).

Sterilized sorghum grains were inoculated with a 10‐mm disk of colonized agar culture. The inoculated grains were then incubated at 25°C for 15 days, allowing for the complete colonization of all grains by fungal mycelium. Subsequently, the resulting spawn, comprising sorghum grains fully colonized by mycelium, was used to treat the substrates (van Kuijk et al., 2017).

### 2.3 Experimental setup

Lignocellulosic materials were subjected to fungal treatment using the fungal spawn. At the start of the experiment, 11 ± 0.2 w/w % DM (dry matter) of prepared spawn consisting of colonized sorghum grains was added to the substrate placed in the bottles. The weights of the substrate and spawn were accurately measured. The spawn was mixed aseptically with the substrate using sterile spoons and tweezers to ensure even distribution. The inoculated substrates were then placed in 250 mL glass bottles and incubated at 26°C under 70%–80% relative humidity (Chaitanoo et al., 2021; van Kuijk, del Río, et al., 2016). All fungal treatments were performed simultaneously and repeated three times at different periods.

### 2.4 Sampling techniques and procedures

At 4‐week intervals during the 12‐week treatment period, a 250 mL glass bottle of each fungus–substrate combination was removed from the incubator. After manual mixing, the treated substrate was dried at 60°C for 72 h, then ground through a 1‐mm sieve and subjected to chemical (e.g., fibers), physical (cellulose crystallinity), and biological (digestibility) analyses.

The choice of treatment duration—4, 8, and 12 weeks—was a critical aspect of our study design. It allowed us to gain insights into the short‐term, mid‐term, and long‐term effects of fungal strain treatment on these agro‐industrial by‐products.

### 2.5 Physical, chemical, and biological analyses

#### 2.5.1 Fiber composition analysis

Fibers were analyzed according to the Van Soest method (Van Soest et al., 1991). Acid detergent lignin (ADL) was defined as L, while C was determined as the difference between acid detergent fiber (ADF) and ADL. The variation or change in C and L (C.var and L.var) was calculated to evaluate the changes in the fibers compared to the control (Benaddou et al., 2023a, 2023b). L.var was calculated using the following formula (Equation 1):

(1) $$\;L.\mathrm{var}=\left(\left({\mathrm{ADL}}_{t}-{\mathrm{ADL}}_{c}\right)/{\mathrm{ADL}}_{c}\right)*100$$

where ADL_c_ is the ADL of the control within the same treatment duration and ADL_t_ is the ADL of the treated sample with one of the fungi. The control values were then excluded before statistical analysis.

C.var was calculated using the following formula to determine the change of C during treatment (Equation 2):

(2) $$C.\mathrm{var}=\left(\left(\left({\mathrm{ADF}}_{t}-{\mathrm{ADL}}_{t}\right)-\left({\mathrm{ADF}}_{c}-{\mathrm{ADL}}_{c}\right)\right)/\left({\mathrm{ADF}}_{c}-{\mathrm{ADL}}_{c}\right)\right)*100$$

where ADF_c_ and ADL_c_ are the ADF and ADL of the control within the same treatment duration and ADL_t_ and ADF_t_ are the ADL and ADF of the sample treated with one of the fungi.

#### 2.5.2 In vitro digestibility assessment

For in vitro true digestibility (IVTD) measurements, rumen contents were collected from four Simmental bulls after slaughter at a local abattoir. The rumen contents (fluid and fibrous mat) were promptly transported to the laboratory in thermos containers, maintaining a temperature of 39.0°C. Upon arrival, the rumen contents were homogenized, filtered through two layers of cheesecloth under a carbon dioxide (CO_2_) atmosphere, and incubated using an ANKOM DAISYII incubator. This specialized incubator is equipped with four rotating digestion jars and ensures consistent heat (39.0°C) and agitation. Each incubation jar received 1600 mL of buffer solution and 400 mL of rumen fluid as inoculum, along with 25 filter bags containing the samples (Ankom F57) (Gulecyuz, 2017).

Approximately 0.5 ± 0.05 g of the sample was placed into each filter bag. Subsequently, each filter bag was introduced into its respective incubation cylinders containing the solution. Before sealing, the cylinder was aerated with CO2 for 40 s. The sealed cylinders were then placed into the incubator for a 48‐h incubation period. Following incubation, the solution was drained, and the filter bags were cleaned with water and subsequently dried at 60°C for 72 h. The NDF determination procedure was then followed to eliminate the remaining cell solubles (Abrahamsen et al., 2021; Tilley & Terry, 1963). The in vitro true digestibility (IVTD) of both treated and untreated samples was calculated using the formula (Equation 3):

(3) $$\text{IVTD}\;\left(\%\right)=100-\left(\frac{W3-\left(W1\times C1\right)}{W2}\right)$$

where: W1: Weight of the filter bag, W2: Weight of sample DM, W3: Final weight after NDF analysis, and C1: blank bag correction.

IVTD.var was used to assess the change (in %) in IVTD between the treated and control samples.

#### 2.5.3 Fungal saccharification

In this study, we employed the 3,5‐dinitrosalicylic acid (DNSA) method to assess fungal saccharification, which is essential for evaluating the efficiency of fungal enzymes in converting complex carbohydrates into valuable reducing sugars (RS) (Boshagh, 2021. RS variation (RS.var) was calculated using the formula (Equation 4):

(4) $$\mathrm{RS}.\mathrm{var}=\frac{\left({\mathrm{RS}}_{t}-{\mathrm{RS}}_{c}\right)}{{\mathrm{RS}}_{c}}*100,$$

where RS.var represents the percentage change in reducing sugars, ${\mathrm{RS}}_{t}$ is the reducing sugars content in the treated substrate after 12 weeks, and ${\mathrm{RS}}_{c}$ is the reducing sugars content in the control substrate.

#### 2.5.4 Quantifying crystallinity index

In this study, we used X‐ray diffraction (XRD) to determine cellulose crystallinity through the Segal crystallinity index (CI). Diffractograms were meticulously recorded over the range of 10° to 30° at a scan rate of 2 per minute. The CI was calculated by evaluating the height ratio between the intensity of the crystalline peak (I002 – *I*_am_) and the total intensity (I002). This calculation was performed after subtracting the background signal measured without cellulose (C) using the empirical equation developed by Segal et al. (1959) and further discussed by Varma et al. (2019) (Equation 5):

(5) $$\mathrm{CI}=\left(\frac{I_{002}-I_{\mathrm{am}}}{I_{002}}\right)*100$$

Additionally, the Bragg equation was employed to determine the *d*‐spacing (interplanar spacing) as follows (Equation 6):

(6) $$d=\frac{\mathrm{n\lambda}}{2\text{sinθ}}$$

Here, ‘*λ*’ represents the incident X‐ray wavelength (1.5406 Å), ‘θ’ signifies the peak position in radians, ‘*n*’ corresponds to the diffraction order, and ‘*d*’ denotes the *d*‐spacing measured in Ångströms (Å).

To calculate the crystallinity index (CI) variation (CI.var) after 12 weeks, we used the following formula (Equation 7):

(7) $$\mathrm{CI}.\mathrm{var}=\frac{\left({\mathrm{CI}}_{t}-{\mathrm{CI}}_{c}\right)}{{\mathrm{CI}}_{c}}*100$$

where CI.var represents the percentage change in CI after 12 weeks. CI_t_ is CI of the treated substrate after 12 weeks. CI_c_ is CI of the control (untreated) substrate.

#### 2.5.5 Dry matter, ash, crude protein (CP), ether extract (EE), and nonfiber carbohydrates (NFC) content analysis

Dry matter (DM) was determined at 70°C for 48 h (Benjamim da Silva et al., 2022). Ash content was determined by combustion in a muffle furnace at 550°C for 6 h (Liu, 2019).

The crude protein (CP) and ether extract (EE) contents were determined, as proposed by the Association of Official Analytical Chemists (AOAC) (1990). NFC was determined using Equation 8 (Mertens, 1997):

(8) NFC%=100%−NDF%−CP%−EE%−Ash%

where NDF refers to neutral detergent fiber, EE refers to ether extraction, and CP stands for crude protein.

### 2.6 Measuring enzymatic activity

Maize was dried at 60°C for 72 h before being milled and passed through a 1‐mm screen. Enzymatic extraction was carried out using a modified version of the method described by Rodrigues et al. (2008). Laccase activity was determined using 2,2′‐azino‐bis (3‐ethylbenzthiazoline‐6‐sulfonic acid) (ABTS) as substrate, and lignin peroxidase assay was determined using the azure B dye as a substrate. Manganese peroxide (MnP) activity was measured by monitoring the oxidation of Mn^2+^ to Mn^3+^ in 0.11 M sodium lactate, and the enzyme activities were expressed in IU/mL. Cellulase activity was determined, according to the Ghose method (Ghose, 1987).

It should be noted that all the activities detected at the beginning were checked at the beginning of each treatment period (at the beginning, after 4 weeks of treatment, and after 8 weeks of treatment) without quantification.

### 2.7 Statistical methods and analysis

The experiments were performed in triplicate, and the sources of variation were identified as fungal species, substrates, blocks, and treatment duration. Normality and homogeneity of the variances were tested using the Shapiro–Wilk test and Bartlett's test, respectively. To evaluate the combined effect of fungus, substrate, and treatment duration on biomass degradation measurements (C.var, L.var, and IVTD.var), a three‐way analysis of variance (ANOVA) was performed using R software (Alkarkhi & Alqaraghuli, 2020). Tukey's test was used for multiple comparisons at a significance level of 0.05. To study the associations between biomass changes (C.var, L.var, and IVTD.var) and the different fungi, substrates, and treatment times, a principal component analysis (PCA) was carried out by R. The alluvial graph was plotted using Origin software to visualize the transitions and connections between the different variables (fungus, substrate, and treatment duration). The alluvial plot represented only positive observations (C.var > 0, L.var > 0, and IVTD.var > 0). The stacked bars represented variables, and the segments represented the frequency observations (*F*) in the data frame that belonged to that level. In other words, *F* represented the occurrence of factors (fungi, substrates, or duration treatment) in cellulose, lignin, or digestibility change. The colored streams between the stacked bars represented a group of observations corresponding to the value for each variable indicated by the stream, and the thickness of the stream indicated how many observations belonged to that group.

To create the clustergram, we utilized the OriginPro software after standardizing the variables (L.var, C.var, and IVTD.var). The distance type employed was the Pearson correlation. The row names are composed successively of the fungus name, followed by the substrate treated by this fungus, and subsequently, the treatment duration (4, 8, or 12 weeks). For example, ‘*F. oxysporum‐OP‐8w*’ signifies olive pomace treated by *F. oxysporum* for 8 weeks.

## 3 RESULTS

### 3.1 Fungal strains and substrate characteristics

The physicochemical characteristics of the substrates are shown in Table 1. The analysis revealed significant differences in fiber, CI, and RS among the different lignocellulosic biomass samples. As shown in Table 2, cellulase and ligninase activities were present in all fungi regardless of the selection medium, except for *F*. sp. and *C. viridescens*.

**TABLE 1:** Chemical and physical properties of lignocellulosic materials.

Items	Substrates		
	OP	CW	WS
DM (%)	66.7 ± 1.2^a^	59.4 ± 1.9^b^	79.2 ± 2.0^c^
OM (%)	98.4 ± 2.0^a^	98.4 ± 1.4^a^	90.5 ± 2.1^b^
NDF (%)	83.2 ± 1.4^a^	75.4 ± 1.7^b^	84.5 ± 2.2^a^
Cellulose (%)	34.7 ± 2.1^a^	35.7 ± 1.9^a^	49.3 ± 1.9^b^
Lignin (%)	21.9 ± 1.0^a^	18.4 ± 0.1^a^	11.4 ± 2.4^b^
Hemicellulose (%)	26.5 ± 1.4^a^	21.3 ± 3.2^a^	23.8 ± 2.5^a^
Crude protein	02.3 ± 0.2^a^	01.3 ± 0.2^b^	01.4 ± 0.1^b^
CI (%)	41.2 ± 3.0^a^	54.5 ± 2.5^b^	34.0 ± 2.7^a^
D (nm)	00.81 ± 0.01^a^	00.799 ± 0.02^a^	00.792 ± 0.01^b^
pH	04.2 ± 0.4^a^	06.0 ± 0.7^b^	05.7 ± 0.3^b^
RS (%)	37.3 ± 5.3^a^	29.3 ± 6.3^b^	37.5 ± 4.4^a^
EE (%)	4.3 ± 1.2^a^	0.4 ± 0.5^a^	1.03 ± 0.5^a^
NFC (%)	8.6 ± 1.2^a^	21.3 ± 1^b^	3.77 ± 1.25^c^

*Note*: Means (±SD), within the same row, lacking a common superscript differ (*α* = .05).

Abbreviations: CI, crystallinity index; C, cellulose; CW, cedar sawdust wood; D, average of *d*‐spacing; DM, Dry matter (just before treatment); EE, ether extraction; L, lignin; NDF, neutral detergent fiber; NFC, nonfiber carbohydrate; OM, organic matter; OP, olive pomace; RS, reducing sugar; WS, wheat straw.

**TABLE 2:** Enzyme activities in submerged fermentation after 10 days.

Enzymes	Cellulase activity (IU mL^−1^)		Ligninase activity (IU mL^−1^)		
	Endoglucanase	β‐Glucosidase	Laccase	Lignin peroxidase	Manganese peroxidase
*P. chrysogenum*	ND^a^	0.68 ± 0.1^a^	2.30 ± 0.1^a^	ND^a^	3.30 ± 0.1^a^
*F*. sp.	ND^a^	ND^d^	ND^c^	ND^a^	0.60 ± 0.1^c^
*F. solani*	0.64 ± 0.1^b^	1.05 ± 0.1^b^	1.04 ± 0.1^b^	ND^a^	ND^b^
*F. oxysporum*	ND^a^	2.93 ± 0.2^c^	ND^c^	4.47 ± 0.1^b^	ND^b^
*C. viridescens*	7.75 ± 0.2^c^	ND^d^	ND^c^	ND^a^	ND^b^
*P. crustosum*	2.12 ± 0.1^d^	ND^d^	0.23 ± 0.1^d^	0.12^c^	ND^b^

*Note*: Means (±SD), within the same column, lacking a common superscript differ (*α* = .05).

Abbreviations: ND, not detected.

### 3.2 Effect of fungi

The performance of selected fungal strains in degrading lignocellulosic substrates and improving nutritional value was assessed in this study. The degradation efficiency of L and the release of digestible nutrients, such as C, were evaluated for each strain.

**FIGURE 1:**
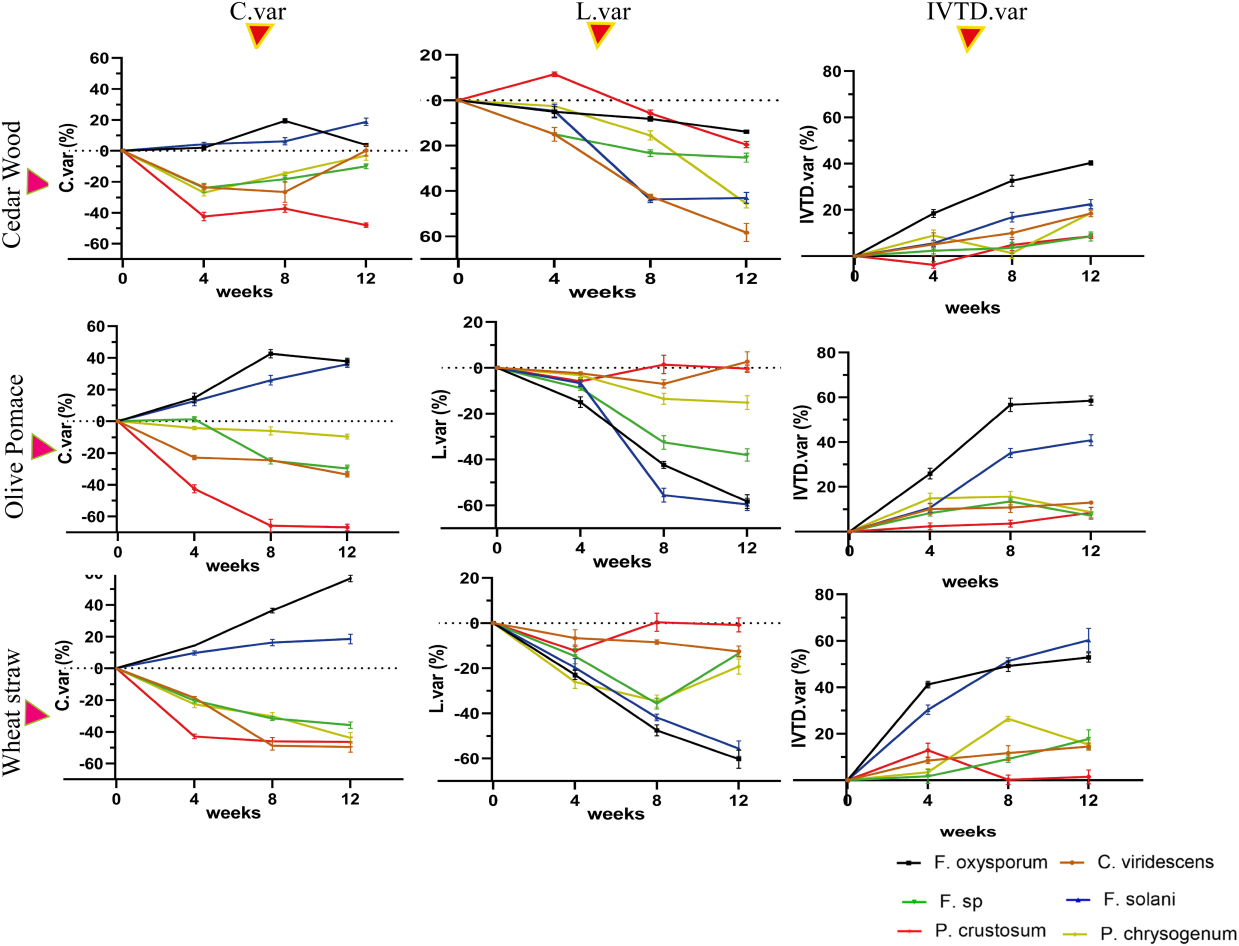
Evolution of fibers (C.var and L.var) and digestibility (IVTD.var) after treatment of substrates (CW, cedar wood; OP, olive pomace; WS, wheat straw) by fungi for 4, 8, and 12 weeks.

**FIGURE 2:**
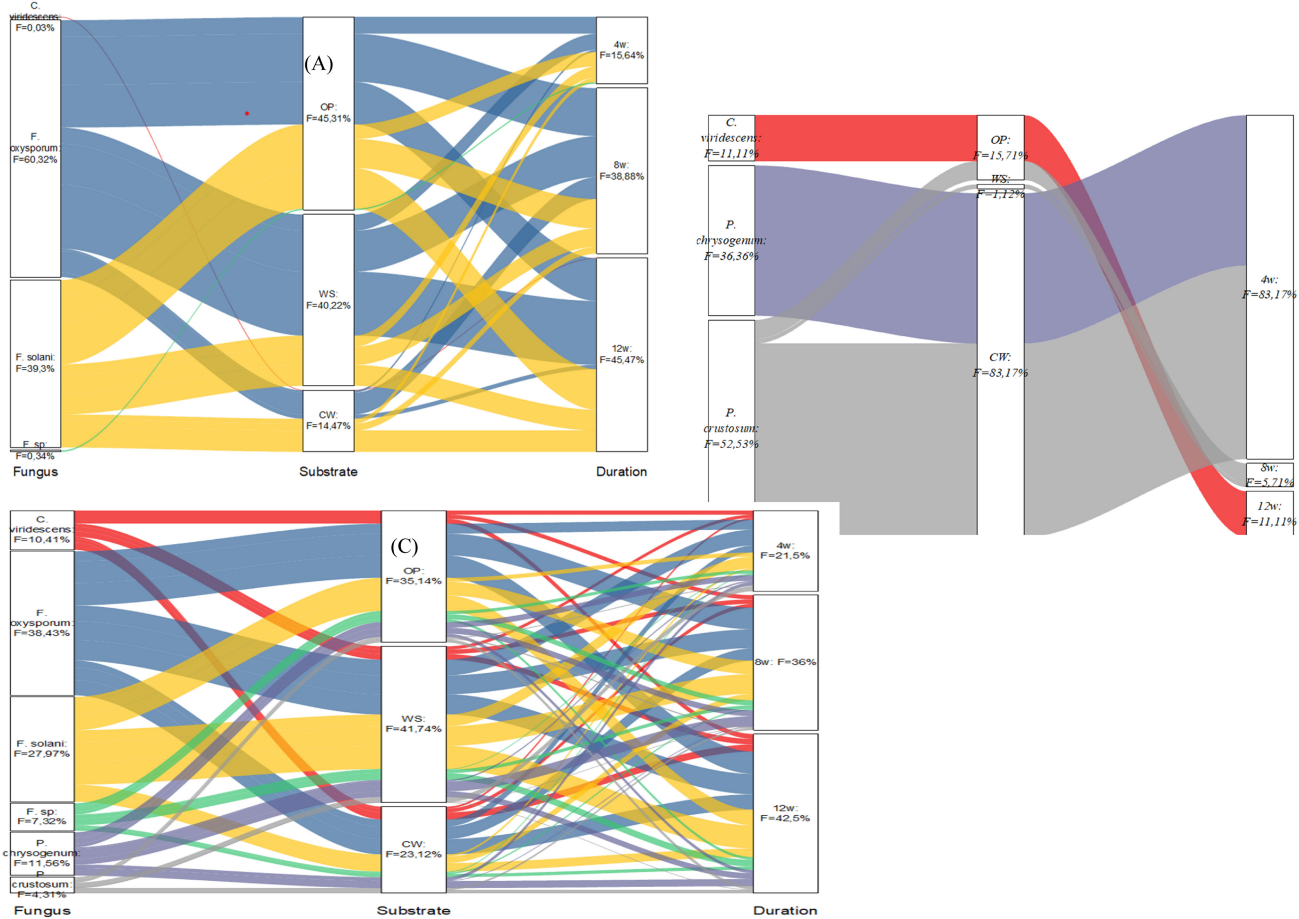
Visualizing the effectiveness of fungal treatment on the treated substrate over time using an alluvial diagram with positive observations. (a) Visualization of C.var; (b) visualization of L.var; and (c) visualization of IVTD.var. CW, cedar wood sawdust; *F*, frequency of observation; OP, Olive pomace; WS, wheat straw.

**FIGURE 3:**
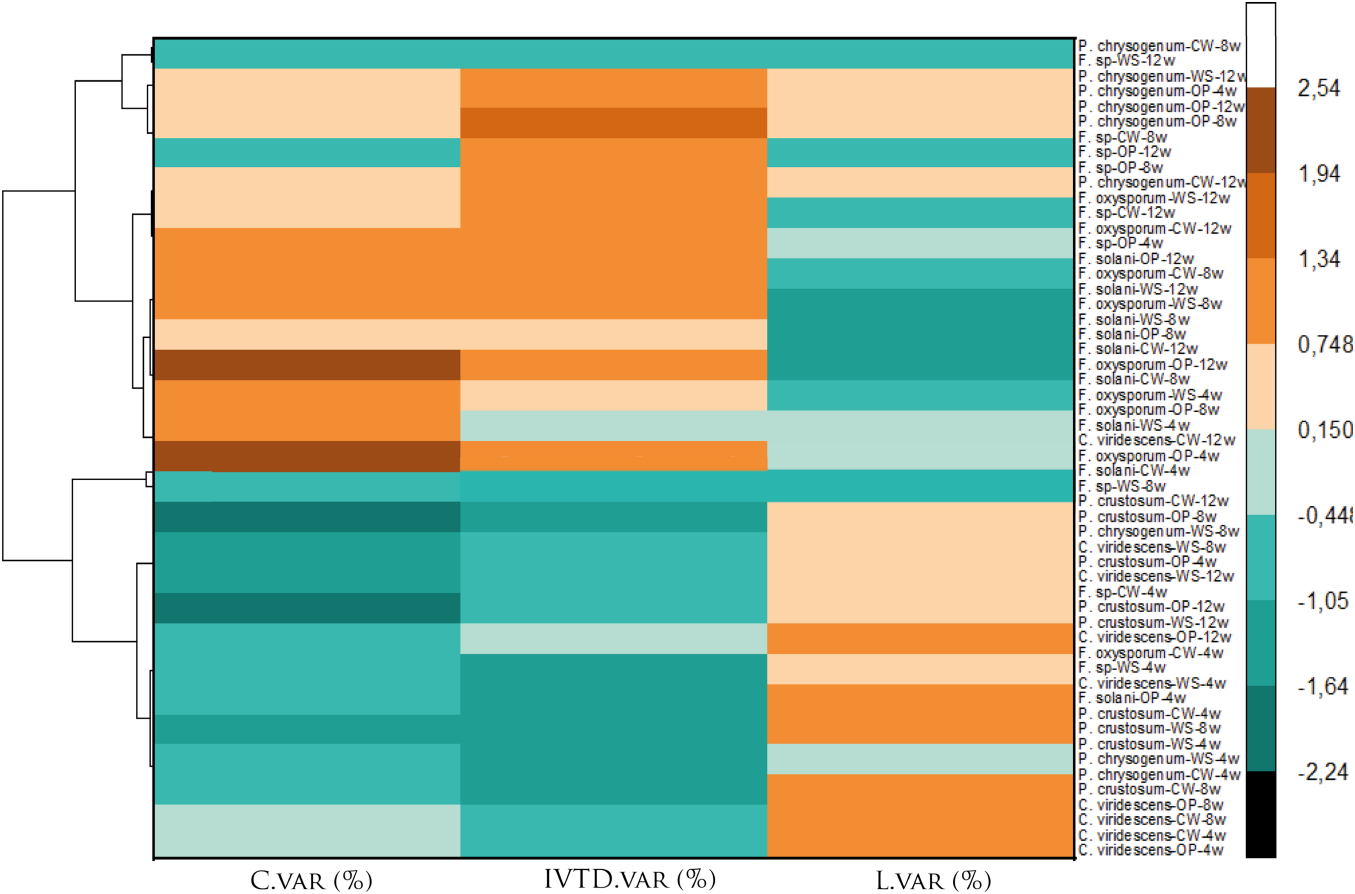
Clustergram of fungal treatment effects on different substrates over varied durations.

**FIGURE 4:**
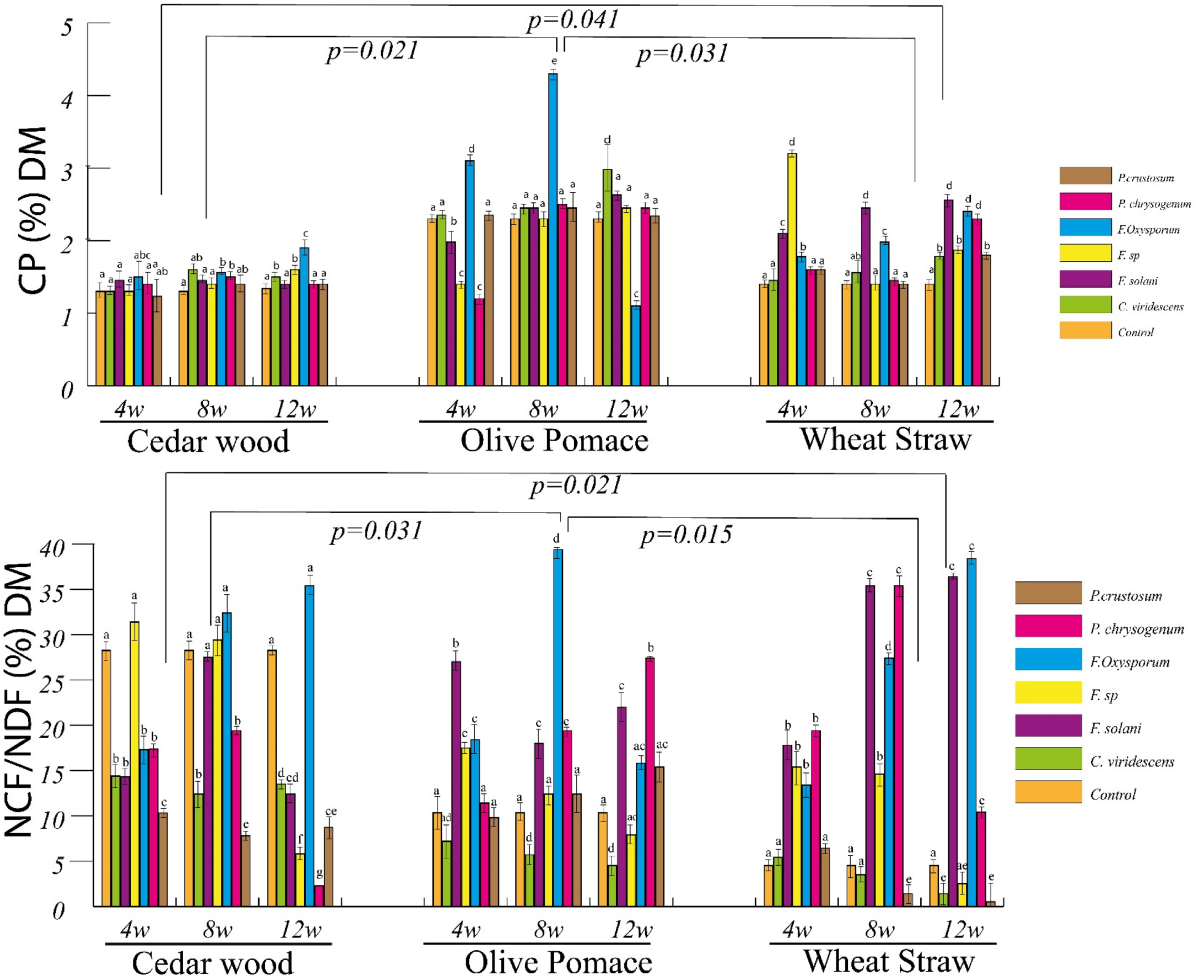
Changes in the NFC/NDF ratio and crude protein content of lignocellulosic substrates (CW, cedar wood sawdust; OP, olive pomace; WS, wheat straw) treated by various fungal strains (*F. solani*, *F. oxysporum*, *F*. sp., *P. chrysogenum*, *P. crustosum*, and *C. viridescens*) over a 12‐week duration. ^abc^Difference in superscripts in the same substrate indicates significance at *p* < .05.

Our results demonstrated that the different fungi used had a significant effect on several parameters, including C, C.var, L, L.var, IVTD, and IVTD.var, NFC, and CP (*p* < .001), but EE wasn't significantly different (*p* = .78). As presented in Figures 1, 2, and 3, *P. crustosum* showed the highest C degradation (C.var = −49%) and a reduced ratio of NFC/NDF. Conversely, *F. oxysporum* and *F. solani* conserved larger amounts of C from the substrates, with mean values of 25% and 16% for C.var and 60.3% and 39.3% for *F*, respectively, and increased NFC/NDF ratio (Figure 4). However, *F*. sp., *C. viridescens*, and *P. chrysogenum* showed slight conservation of cellulose (mean C.var of −22%) and maintained NFC/NDF. All fungi increased CP compared to the control, but only *P. crustosom* and *F. solani* showed a significant increase.

Regarding L degradation, *F. oxysporum* and *F. solani* were the most efficient, removing more than 40% of the L, followed by *F*. sp. with 25% compared to the control. Contrarily, *P. crustosum* and *C. viridescens* did not show significant degradation of L from any of the substrates. They increased the amounts of lignin from the substrates (*F* = 52.52% and *F* = 36.32%, respectively). *P. chrysogenum* showed insignificant L reduction from OP and CW, but significant decomposition from WS (L.var = −26.65 ± 7.1%).

In terms of digestibility, *F. oxysporum* and *F. solani* emerged as the best fungi that significantly improved digestibility compared to the other fungi (mean of IVTD.var was 41.71 ± 13.6% and 30.35 ± 17.9%, respectively). *C. viridescens* and *P. chrysogenum* slightly increased digestibility for all substrates (the mean of IVTD.var was 11.90 ± 5.8% for both). *F*. sp. and *P. crustosum* showed insignificant improvement in digestibility (Figures 1, 2, and 3). Of the total increase in digestibility, *F. oxysporum* and *F. solani* accounted for a large proportion (*F* was 28.5% for both).

### 3.3 Effect of substrate

The study investigated the effect of substrates, including WS, CW, and OP, on fungal treatment. First, there were significant differences in physicochemical composition (dry matter (DM), fibers, pH, CI, and RS) among the three substrates before treatment. The DM content varied with WS having the highest value (79.22 ± 2.00%), followed by OP (66.74 ± 1.20%) and CW (59.41 ± 1.90%). OP and CW had similar percentages of C and L (about 35% and 20%, respectively), whereas WS had a higher percentage of C (49.35%) and a lower percentage of L (11.4 ± 2.40%).

Second, after treatment, Figures 1 and 2 show that the substrates differed significantly in their IVTD.var, C.var, L.var, and ADF (*p* < .001) (Table 3). As shown in Figure 2, the study revealed that lignin and cellulose of CW were highly resistant to fungal attack followed by OP and WS. The NFC/NDF ratio of WS increased more than those of OP and CW. On the other hand, CP increased more in WS and OP than in CW. The digestibilities of treated WS and OP were significantly improved (the mean of IVTD.var was 22.65 ± 17% and 19 ± 17.26% respectively). Among the increased total digestibility (IVTD.var > 0) of treated substrates, the digestibility of WS makes up a large proportion (*F* = 41.75), followed by OP (*F* = 35.14%) and CW (*F* = 23.3%).

**TABLE 3:** Change in crystallinity index (CI.var) and reducing sugars (RS.var) in fungus‐treated substrates after 12 weeks.

Substrates	Cedar wood		Olive pomace		Wheat straw	
	CI.var (%)	RS.var (%)	CI.var (%)	RS.var (%)	CI.var (%)	RS.var (%)
*C. viridescens*	−42.5 ± 1.0^a^	−48.3 ± 0.01^a^	−26.6 ± 0.6^b^	−08.3 ± 3.5^b^	−34.0 ± 2.3^C^	−45.4 ± 6.5^a^
*P. crustosum*	−01.5 ± 2.0^a^	−14.7 ± 1.1^a^	15.6 ± 1.3^b^	−26.6 ± 4.1^b^	05.4 ± 6.4^c^	−34.1 ± 6.8^b^
*F. oxysporum*	−07.7 ± 5.0^a^	20.3 ± 1.9^a^	−10.3 ± 3.40^b^	32.1 ± 5.2^b^	−14.7 ± 2.7^c^	77.3 ± 0.1^c^
*F. solani*	−39.7 ± 1.0^a^	34.2 ± 0.6^a^	−10.6 ± 1.1^b^	40.3 ± 3.1^b^	−29.3 ± 2.2^c^	58.7 ± 3.2^c^
*F. chrysogenum*	05.5 ± 0.9^a^	−37.5 ± 5.1^a^	04.9 ± 2.2^a^	04.9 ± 2.2^a^	−27.5 ± 1.8^b^	−04.60 ± 1.6^c^
*F*. sp.	−14.8 ± 4.0^a^	05.2 ± 9.1^a^	−19.3 ± 4.4^b^	01.4 ± 6.7^b^	−15.2 ± 4.8^b^	61.9 ± 12.8^c^

*Note*: Means (±SD), within the same row and the same parameter, lacking a common superscript differ (*α* = .05).

### 3.4 Effect of biomass Treatment duration

Lignin degradation (L.var) and digestibility improvement (IVTD.var) differed significantly (*p* < .001) between treatment duration. However, C.var was not significant across treatment durations and averaged −17 ± 26%. L.var averaged −12 ± 15%, −27 ± 15%, and −33 ± 21% during 4, 8, and 12 weeks of incubation, respectively. IVTD.var averaged 22 ± 20%, 30 ± 23%, and 40 ± 20% during 4, 8, and 12 weeks, respectively. Cellulose degradation occurred significantly within 4 weeks of incubation for most fungi, while lignin degradation occurred after 8 weeks (Table 3).

### 3.5 Interaction fungus × substrate × treatment duration

**FIGURE 5:**
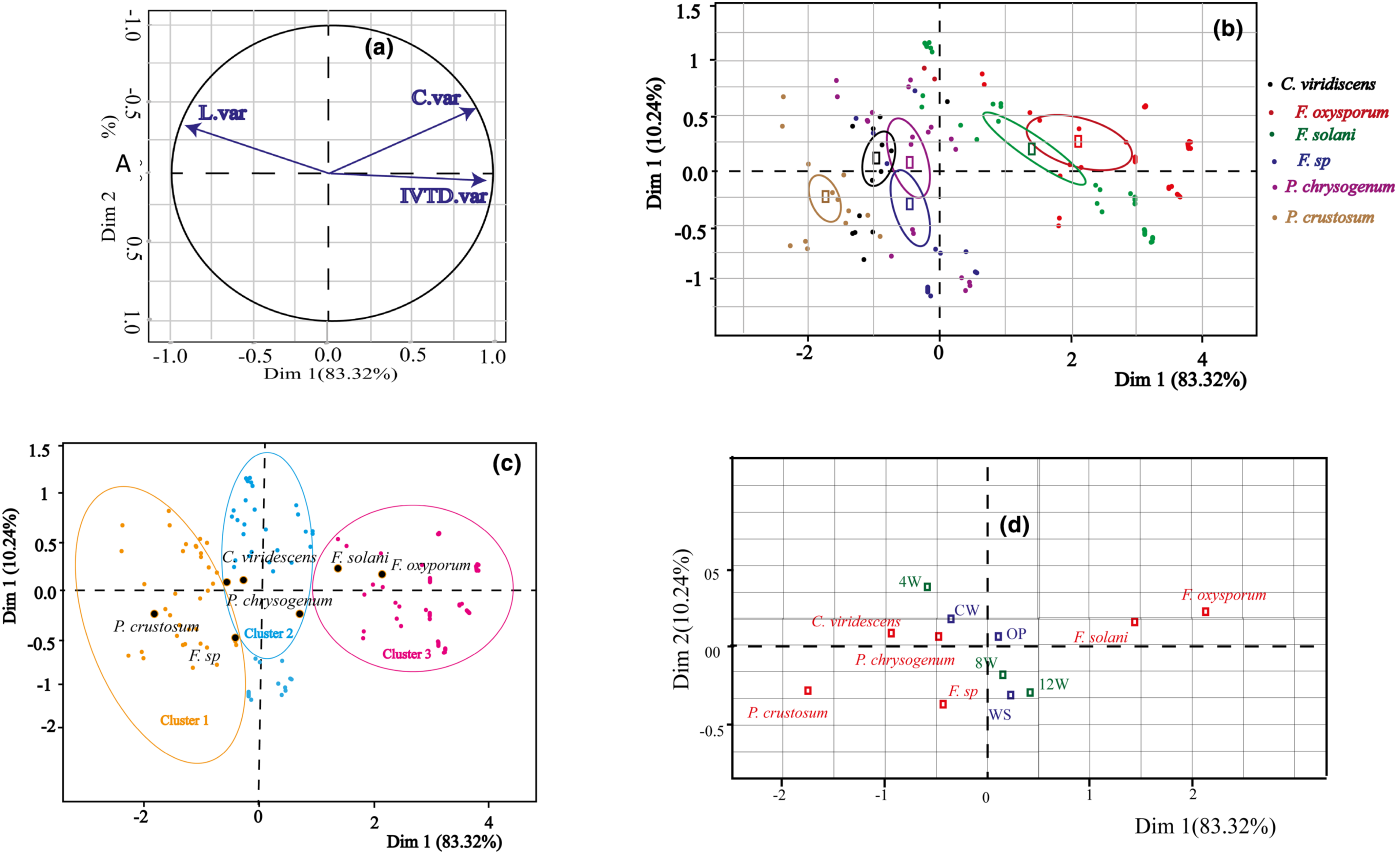
Principal component analysis (PCA). (a) Variables factor map (PCA); (b) Individuals factor map (PCA); (d) Qualitative factor map (PCA); and (c) Ascending Hierarchical Classification of the individuals. The labeled factors, variables, and individuals are those the best shown on the plane.

The enhanced nutritional value of the treated biomass is typically associated with notable increases in cellulose availability, concurrent reductions in lignin content, and a substantial elevation in overall digestibility. As presented in Figure 5, *F. solani* and *F. oxysporum* significantly improved the nutritional value of the treated substrates compared to the other fungi and the control after up to 8 weeks of incubation. However, it should be noted that the speed of this improvement was very high in the case of WS, followed by OP, but this improvement was low in CW. After 8 weeks, *F. oxysporum* was found to have degraded cellulose in CW and OP but continued to increase cellulose in WS. For *F. solani*, a greater release of cellulose was observed in the CW after 8 weeks of incubation. There was no significant change in lignin up to 4 weeks of treatment compared with the control in all fungi. *P. crustosum* and *C. viridescens* had no significant effect on the change in percentage of lignin in OP and WS, but *C. viridescens* reduced lignin in CW by more than 50% compared with the control after 12 weeks of incubation. *P. chrysogenum* degraded WS lignin by 25% relative to the control during 8 weeks of treatment, but after this duration, the change in lignin was negligible. In terms of digestibility, *F. oxysporum* and *F. solani* increased the digestibility of all substrates exponentially over time. *P. chrysogenum* slightly (but not more than 20%) increased CW digestibility continuously up to 12 weeks of treatment, while attacking WS lignin only between 4 and 8 weeks.

### 3.6 Variables correlations

**FIGURE 6:**
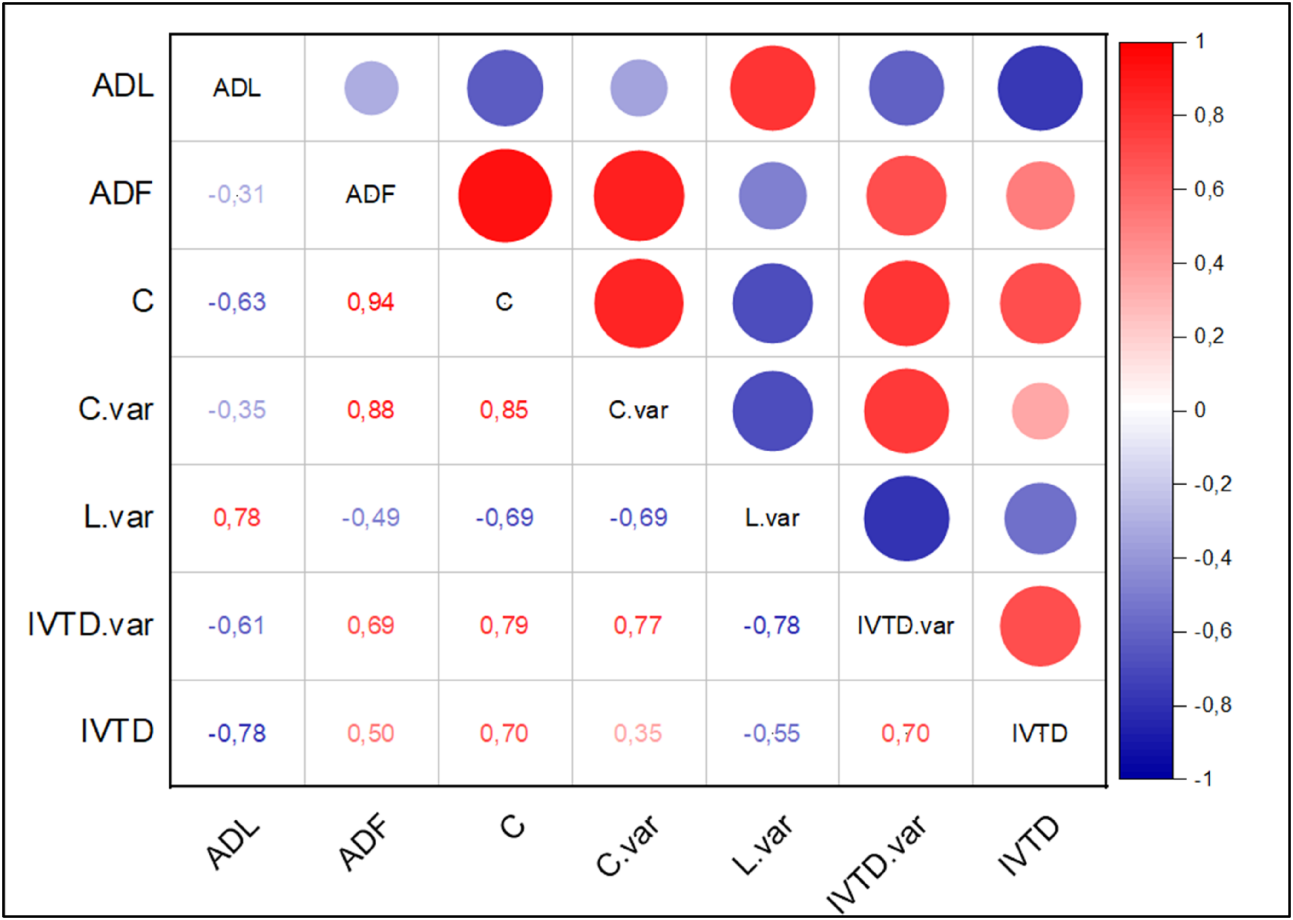
The Pearson correlation between parameters. C, Cellulose; C.var, cellulose variation; IVTD, in vitro true digestibility; L.var, lignin variation.

As depicted in Figure 6, the inertia of the first dimensions indicates whether there are strong relationships between variables and suggests the number of dimensions that should be studied. The total dataset inertia is expressed by the first two dimensions, accounting for 93.56% of the total variability.

Moving on to the hierarchical classification of individuals, the presence of three clusters was revealed:

Cluster 1 consists of individuals (the substrates treated by *P. crustosum* and *C. viridescens*) sharing the following characteristics: High values for the variable L.var and low values for the variables C.var and IVTD.var (variables are sorted from the weakest).
Cluster 2 includes individuals (the substrates treated *by F*. sp. and *P. chrysogenum*) characterized by C.var, L.var, and IVTD.var whose values do not significantly differ from the mean.
Cluster 3 comprises individuals (the samples treated by *F. oxysporum* and *F. solani*, especially the samples of WS and OP incubated for 12 weeks) characterized by: High values for the variables IVTD.var and C.var (variables are sorted from the strongest) and low values for the variable L.var.

The Pearson correlation coefficient (r) between C, C.var, L, L.var, ADL, ADF, IVTD.var, and IVTD is presented in Figure 6. Positive correlations were observed between C and IVTD (*r* = .7), and between C.var and IVTD.var (r = 0.79). In contrast, negative correlations were found between L.var and C.var (*r* = −.69), L.var and IVTD.var (*r* = −.78), and ADL and IVTD (*r* = −.78).

## 4 DISCUSSION

The present study aimed to evaluate the performance of six different fungi in improving the nutritional value of three lignocellulosic substrates (CW, WS, and OP). The fungi were subjected to treatment durations of 4, 8, and 12 weeks to assess their efficacy over a varying duration. Fungi can optimize the nutritional value of lignocellulosic biomass if they improve IVTD, increase cellulose content, reduce crystallinity index, lower lignin levels, enhance crude protein, and improve nonfiber carbohydrate (NFC) and RS content.

### 4.1 Effect of fungi

The selected fungi display different abilities to break down the lignin barrier while demonstrating a different selectivity in their approach to cellulose processing (Figures 1 and 2). Additionally, these fungi showed differences in the release of NFC and the production of CP (Figure 4). Several fungi have developed the production of lignocellulolytic enzymes, creating a synergistic system capable of degrading the primary polymers found in lignocellulosic biomass. These enzymes encompass various categories: (1) ligninolytic enzymes, including laccases, lignin peroxidase, manganese peroxidase, and versatile peroxidase; (2) cellulolytic enzymes, such as endoglucanase, exoglucanase, and β‐glucosidase; and (3) hemicellulolytic enzymes, including endoxylanases, arabinofuranosidase, β‐xylosidases, feruloyl esterase, and others. Together, these enzymes form a synergistic system capable of efficiently degrading lignocellulosic biomass (Saldarriaga‐Hernández et al., 2020). Fungal species exhibiting high selectivity in lignin degradation with minimal impact on cellulose (>20% lignin and <5% cellulose degraded) included *Fusarium solani*, *Fusarium oxysporum*, *F*. sp., and *Penicillium chrysogenum* (Figures 1 and 4, Table 4). These fungi share characteristics with *Ceriporiopsis subvermispora*, *Lentinula edodes*, *Hericium clathroides*, *Pleurotus ostreatus*, and *Pleurotus eryngii* (Labuschagne et al., 2000; Liang et al., 2010; Makkar & Singh, 1992), and *Pleurotus salmoneo‐stramineus* (Bickel Haase et al., 2024).

**TABLE 4:** Comparative analysis of fungal treatment of lignocellulosic substrates.

Fungal strains	Substrates tested	Inoculum concentration	*T* (°C)	Treatment duration (days)	Effect versus control	Reference
*Fusarium oxysporum*	Wheat straw	NS	40	56	Enhance the saccharification of lignocellulose during the simultaneous saccharification and fermentation (SSF) process	Xiros et al. (2009)
	Wheat straw	NS	30	21	The bioconversion yield of hydrolysis was 52% (based on total cellulose content)	Paschos et al. (2015)
	Wheat straw	NS	26	84	Cellulose improvement, lignin degradation, and IVTD improvement up to 56%, 60%, and 52.86%, respectively	This work
*Fusarium solani*	Wheat straw	11w/w%	26	84	Cellulose improvement, lignin degradation, and IVTD improvement up to 18.52%, 56.64%, and 60.33%, respectively	This work
*Penicillium chrysogenum*	Wheat straw	11w/w%	26	84	Cellulose loss, lignin degradation, and IVTD improvement up to 43.85%, 19.27%, and 15.30%, respectively	This work
*Cosmospora viridescens*	Wheat straw	11w/w%	26	84	Cellulose loss, lignin degradation, and IVTD improvement up to 49.62%, 12.57%, and 14.46%, respectively	This work
*Penicillium crustosum*	Wheat straw	11w/w%	26	84	Cellulose loss, lignin degradation, and IVTD improvement up to 46.44%, 0.81%, and 1.4%, respectively	This work
*Lentinula edodes*	Wheat straw	11w/w%	24	84	Degradation of lignin, improvement in cellulose, and an increase in IVGP72 of 81%, 13%, and 23.1%, respectively	van Kuijk et al. (2015)
	Wheat straw	11w/w%	24	56	Decrease in lignin 35% and improvement in cellulose 15%, respectively	Mao et al. (2021)
*Ceriporiopsis subvermispora*	Wheat straw	11w/w%	24	84	Decrease in lignin 83.3% and improvement in cellulose 20.02%, respectively	van Kuijk et al. (2017)
	Wheat straw	NS	24	56	Decrease in lignin 67% and improvement in cellulose 11%, respectively	Mao et al. (2021)
*Irpex lacteus*	Wheat straw	NS	28	84	Lignin loss was 42%	García‐Torreiro et al. (2016)
	Wheat straw	11w/w%	28	56	Improvement in in vitro rumen digestibility was 39%	Niu et al. (2020)
*Inonotus obliquus*	Wheat straw	NS	28	12	Decrease in lignin, hemicellulose, and cellulose up to 72%, 46%, and 55%, respectively	Xu et al. (2017)
*Fusarium oxysporum*	Cedar wood	11w/w%	26	84	Cellulose improvement, lignin loss, and IVTD improvement up to 3.84%, 13.9%, and 40.30%, respectively	This work
*Fusarium solani*	Cedar wood	11w/w%	26	84	Cellulose improvement, lignin loss, and IVTD improvement up 18.78%, 43.03%, and 22.45%, respectively	This work
*Penicillium chrysogenum*	Cedar wood	11w/w%	26	84	Cellulose loss, lignin loss, and IVTD improvement up to 3%, 45.6%, and 18.60%, respectively	This work
*Cosmospora viridescens*	Cedar wood	11w/w%	26	84	Cellulose improvement, lignin loss, and IVTD improvement up to 0.12%, 58.34%, and 18.45%, respectively	This work
*Penicillium crustosum*	Cedar wood	11w/w%	26	84	Cellulose loss, lignin loss, and IVTD improvement, −48%, 19.54%, and 8.54%, respectively	This work
*Pityriasis versicolor*	Beech wood	NS	22	120	Reduction of lignin and cellulose up to 57.4% and 16.7%, respectively	Wang et al. (2013)
*Trametes velutina*	Poplar wood	100 v/w%	28	56	Delignification by 7.2%, whereas both hemicellulose and cellulose were increased by 1% and 6%, respectively	Wang et al. (2013)
*Abortiporus biennis*	Poplar wood	0.32 w/w %	27	30	Degradation of lignin, hemicellulose, and cellulose reached 17.1%, 19.3%, and 7.4%, respectively	Alexandropoulou et al. (2017)
*Pseudotsuga menziesii*	Willow sawdust	0.48 w/w %	27	30	Lignin, hemicellulose, and cellulose loss of 30.5%, 42.4%, and 26.6%, respectively	Alexandropoulou et al. (2017)
*Stereum hirsutum*	Radiata pine	NS	25	21	Loss of lignin at 16%, while both hemicellulose and cellulose at 5% each	Shirkavand et al. (2017)
	Radiata pine	NS			Lignin degradation of 16%, whereas both hemicellulose and cellulose of 9% individually	Shirkavand et al. (2017)
*Trametes versicolor*	Pinus yunnanensis	NS	28	84	Mass loss and crystallinity index up to 10.6% and −14%, respectively	Qi et al. (2023)
	Cunninghamia lanceolate	NS	28	84	Mass loss and crystallinity index up to 31.2% and −18.6%, respectively	Qi et al. (2023)
	Hevea brasiliensis	NS	28	84	Mass loss and crystallinity index up to 19.22% and −2%, respectively	Qi et al. (2023)
	Populus yunnanensis	NS	28	84	Mass loss and crystallinity index up to 81% and 74%, respectively	Qi et al. (2023)
*Gloeophyllum trabeum*	Pinus yunnanensis	NS	28	84	Mass loss and crystallinity index up to 13% and −11%, respectively	Qi et al. (2023)
	Cunninghamia lanceolate	NS	28	84	Mass loss and crystallinity index up to 51.36% and −12.7%, respectively	Qi et al. (2023)
	Hevea brasiliensis	NS	28	84	Mass loss and crystallinity index up to 27.7% and −0.7%, respectively	Qi et al. (2023)
	Populus yunnanensis	NS	28	84	Mass loss and crystallinity index up to 61.20% and −26.8%, respectively	Qi et al. (2023)
*Rhodonia placenta*	Pinus yunnanensis	NS	28	84	Mass loss and crystallinity index up to 035.21% and‐22.3%, respectively	Qi et al. (2023)
	Cunninghamia lanceolate	NS	28	84	Mass loss and crystallinity index up to 38% and −4.3%, respectively	Qi et al. (2023)
	Hevea brasiliensis	NS	28	84	Mass loss and crystallinity index up to 10.25% and −41%, respectively	Qi et al. (2023)
	Populus yunnanensis	NS	28	84	Mass loss and crystallinity index up to 16.46% and −5.6%, respectively	Qi et al. (2023)
*Fusarium oxysporum*	Olive pomace	11w/w%	26	84	Cellulose improvement, lignin loss, and IVRD increase up to 37.83%, 58.64%, and 58.49%, respectively	This work
*Fusarium solani*	Olive pomace	11w/w%	26	84	Cellulose improvement, lignin loss, and IVRD increase up to 35.96%, −59.60%, and 40.84%, respectively	This work
*Penicillium chrysogenum*	Olive pomace	11w/w%	26	84	Cellulose loss, lignin loss, and IVTD increase up to 9.59%, 15.16%, and 8.7%, respectively	This work
*Cosmospora viridescens*	Olive pomace	11w/w%	26	84	Cellulose loss, lignin increase, and IVTD improvement up to 33%, 2.67%, and 12.85%, respectively	This work
*Penicillium crustosum*	Olive pomace	11w/w%	26	84	Cellulose loss, lignin increase, and IVTD improvement up to 66.83%, 0.35%, and 8.4%, respectively	This work

Abbreviation: NS, Not specified.

Based on the PCA (Figures 5 and 6), the fungi used in our study can be classified into three distinct groups. The first group comprises *P. crustosum* and *C. viridescens*, the second group consists of *F*. sp. and *P. chrysogenum*, and the third group is composed of *F. oxysporum* and *F. solani*. This classification suggests that each group, consisting of two fungi, demonstrates a notable level of consistency in their performance when compared to the overall mean values of C.var, L.var, and IVTD.var, NCF/NDF, and CP. The minimal deviations from the mean values across these parameters indicate that the fungi within each group may exhibit relatively balanced and consistent enzymatic activity in cellulose and lignin degradation, as well as in enhancing overall digestibility. It is noteworthy that the enzyme activities and their effects on substrate composition and treatment duration differed from those observed in other studies (Table 4).

The first group (*P. crustosum* and *C. viridescens*) exhibited limited lignin degradation, as indicated by the high value of L.var, which was lower than that observed in *Lentinula edodes* (van Kuijk et al., 2015) (Table 4), suggesting that the targeted fungi did not effectively break down lignin in the lignocellulosic materials possibly due to the absence of or low ligninolytic activity (Table 2). Despite the limited lignin degradation, this group demonstrated a remarkable capacity for cellulose degradation, as evidenced by the low value of C.var, exceeding that of *Pseudotsuga menziesii* (Alexandropoulou et al., 2017). This implies that the fungi in this group modified the lignin structure rather than removing it. This modification allowed them to access the cellulose and hydrolyze it into nonfiber carbohydrates (NFC), especially RS through the action of endoglucanases (Table 2). However, the low NFC and RS content in the substrate treated by these fungi indicates that the released NFC was not primarily utilized for enhancing nutritional parameters but rather converted into secondary metabolites, such as CO_2_ (Ma et al., 2020). Additionally, the limited in vitro true digestibility (IVTD) (Figure 1) observed in this group suggests that despite their proficiency in cellulose degradation, the overall digestibility of the treated substrate remained restricted, possibly due to the redirection of NFC and RS toward secondary metabolic processes.

The second group, comprising *F*. sp. and *P. chrysogenum*, exhibited C.var, L.var, and IVTD.var, CI.var, SR.var, NFC/NDF ratio, and CP values that did not significantly deviate from the mean, indicating a modest improvement in nutritional value. However, this improvement appeared to be substrate‐dependent (Figures 1, 2 and 4). *F*. sp. and *P. chrysogenum* demonstrated moderate activities of laccase and/or manganese peroxidase (Table 2). The laccase and manganese peroxidase system is known for its effectiveness in degrading lignin Chen et al. (2012). This enzymatic system is highly efficient in the oxidation of both phenolic and nonphenolic components of lignin (Debnath & Saha, 2020; Manyapu et al., 2022). Moreover, *P. chrysogenum* exhibited β‐glucosidase activity. β‐glucosidases form a heterogeneous group of hydrolytic enzymes with significant biological importance, acting on various substrates (Mól et al., 2023). Despite the lack of endoglucanase and β‐glucosidase activity measured in *F*. sp., it was able to degrade cellulose, suggesting that this fungus possesses other cellulase enzymes that are not measured, such as endo‐1,4‐β‐glucanase (Erkanli et al., 2024), or the cellulose was degraded by a chemical process after the reduction of lignin. The enhancement of RS, NFC/NDF ratio, and CP in the substrate treated by *F*. sp. and *P. chrysogenum* suggests that these fungi convert cellulose into sugar or utilize this sugar to produce CP. All these factors contribute to improving the nutritional quality, although the enhancement is moderate.

The third group, consisting of *F. oxysporum* and *F. solani*, successfully reduced lignin content, elevated cellulose levels, and significantly improved overall digestibility (indicated by a high value of IVTD.var) of all substrates compared to the two other groups after 12 weeks of incubation. Additionally, this group improved NFC/NDF ratio, RS, and CP, signifying that these fungi were efficient and potent in enhancing nutritional value. It is noteworthy that *F. oxysporum* and *F. solani* demonstrated a lower capacity to degrade wheat straw lignin compared to *Ceriporiopsis subvermispora* and *Lentinula edodes*, as reported by van Kuijk, Sonnenberg, et al. (2016b) and Madadi (2017). On the contrary, *F. oxysporum* and *F. solani* exhibited a higher lignin degradation capacity for wheat straw compared to *Pleurotus ostreatus*, where the lignin reduction did not exceed 34% after 8 weeks of incubation, as reported by Saravanan et al. (2023).

### 4.2 Effect of substrate

The lignocellulosic substrate tested in this study is characterized by a high content of lignified plant cell walls, which includes not only lignin but also hemicellulose and cellulose. Additionally, this lignocellulosic biomass contains only small amounts of ash, proteins, and nonfiber carbohydrates (NFC) (Table 1). The lignin content and composition vary among different feed ingredients, including residues of wheat, rice, corn, oil palm, cocoa, bamboo, sugarcane, water hyacinth, cedar, birch, and spruce (van Kuijk, del Río, et al., 2016), as well as olive pomace (Benaddou et al., 2023b).

Following a 12‐week treatment, substantial variations in IVTD.var, C.var, L.var, and ADF were noted across the substrates (*p* < .001). Notably, the nutritional value of treated wheat straw (WS) exhibited a better improvement than that of cedar wood (CW). This difference can be attributed to various factors. First, the relatively short length of straw fiber cells plays a role, with wheat straw fiber cells averaging 1.18 mm in length and 13.60 μm in width (Singh et al., 2011), while softwood tracheids are approximately 3 mm in length and 20–35 μm in width. Additionally, the cellulose crystallinity index (CI) was higher in CW (CI = 54.5 ± 2.5%) compared to OP (CI = 41.2 ± 3.0%) and WS (CI = 33.4 ± 2.7%). The highly crystalline cellulose in CW, coupled with its elevated lignin content, may limit accessibility to enzymes, hindering the rate and efficiency of nutritional value improvement. In contrast, the amorphous regions of WS cellulose offer a greater reactive surface area compared to crystalline regions. This enhanced reactive surface area provides more sites for enzyme action, facilitating cellulose degradation (Xu et al., 2019). As a result, WS proved to be more accessible for enzymes of most fungi used, leading to improvements in CP, NFC/NDF ratio, IVTD, and cellulose, thereby enhancing its nutritional value. Our study revealed that when exposed to olive pomace (OP) and cedar wood (CW), *P. chrysogenum* and *F*. sp. showed limited lignin breakdown. In contrast, previous studies with other fungi, such as *Pityriasis versicolor* and *Trametes versicolor*, demonstrated more effective lignin degradation in different wood types like *Pinus yunnanensis* (Qi et al., 2023; Wang et al., 2013). However, the scenario changed when wheat straw (WS) was used with *P. chrysogenum* and *F*. sp. In this case, they exhibited significant lignin breakdown, comparable to the results observed with *Pseudotsuga menziesii* when treated with willow sawdust for 30 days in another study (Alexandropoulou et al., 2017). Even though *F*. sp. can break down lignin, it didn't make the wheat straw easier to digest. This is puzzling. Our study also found that when *F*. sp. broke down lignin, it reduced the digestibility by about 25%. Breaking down lignin may have produced chemical inhibitors that make biomass less digestible, reducing its nutritional value.

Olive pomace (OP) is the solid residue that remains after oil is extracted from olives. These residues include pulp, skin, pit, and a small amount of residual oil. The substrate content provides an ideal environment for fungal growth, facilitating the breakdown of complex components such as lignin and cellulose. Fungi can utilize the carbon sources present in olive pomace and promote the release of enzymes that contribute to the breakdown of lignocellulosic materials. This natural decomposition process, driven by fungal activity, enhances the degradation of lignin and cellulose, ultimately affecting the value of olive pomace for various applications, including animal feed and other industrial processes (Innangi et al., 2017; Lammi et al., 2019; van Kuijk, Sonnenberg, et al., 2016).

Tolerance to variable conditions is important for nutritional value improvement by fungi. A successful fungus must be able to function effectively under different environmental conditions, such as temperature, humidity, pH, etc. In this study, a high pH tolerance was observed in *F. oxysporum*. The performance of *F. oxysporum* was maintained in different substrate pH levels (from 4.2 ± 0.4 to 6.0 ± 0.7). Adaptability to different substrates is also an important aspect. Efficient degradation of different types of substrates, such as wheat straw, cedar sawdust, and olive pomace, is a characteristic of the best performing fungus. According to the results, *F. oxysporum* and *F. solani* are adapted and perform optimally on a wider range of lignocellulosic materials (OP, CW, and WS).

### 4.3 Effect of treatment duration

The selection of treatment durations ranging from 4 to 12 weeks was based on our aim to comprehensively evaluate the efficacy of the fungal treatment on the lignocellulosic substrates. These durations were chosen to capture potential changes in nutritional composition and substrate degradation over a range of time points. There was a significant (*p* < .01) interaction between the fungus and treatment duration for all substrates, indicating that different fungi had varied effects over the treatment durations (Figures 1 and 2). The optimal treatment duration in the fungal treatment process of lignocellulosic biomass to improve its nutritional value as forage is a key element (van Kuijk et al., 2015). In this study, fungal treatment was conducted at different durations to investigate whether lignin degradation shifts to cellulose degradation, leading to the formation of NFC and CP after prolonged treatment (Figure 6). The optimal treatment duration is characterized by a rate of lignin degradation (L.var < < 0), which is essential for improving forage digestibility (Jin & Wei, 2023).

While none of the fungi increased IVTD by more than 20% during the first 4 weeks, and none reduced lignin content by less than 20% during this period, IVTD of WS treated with *F. oxysporum* (*p* = .0023) and *F. solani* (*p* = .014) improved by 30% and 40%, respectively. Between 4 and 8 weeks, IVTD for most fungi decreased, except for *F. oxysporum* and *F. solani*. After 8 weeks, the IVTD of *P. chrysogenum* resumed an increase, suggesting that this duration is optimal for improving the nutritional value of wheat straw by *P. chrysogenum*. As for *C. viridescens*, *P. crustosum*, and *F*. sp., the ideal time for nutritional value improvement is 4 weeks. IVTD of substrates treated with *F. oxysporum* and *F. solani* increased by 60% after 8 weeks and was accompanied by lignin degradation and NFC reduction. Therefore, it is advisable to consider 8 weeks as the optimal duration for enhancing the nutritional value.

The time required for fungal growth, enzyme secretion, and enzyme attack on the substrate varied depending on the fungus and substrate (Figures 2 and 6). In general, this study demonstrated that cellulose degradation was significantly achieved by most fungi within 4 weeks of incubation. In contrast, lignin degradation was significantly achieved after 8 weeks of treatment. This can be explained by the fact that cellulose, a linear glucose polymer, is relatively easier to degrade by cellulolytic enzymes produced by fungi. After 8 weeks of treatment, cellulose and lignin degradation may decrease, which can be explained by the accumulation of metabolites in the culture medium. Some of these metabolites may have inhibitory effects on fungal enzymatic activity, which can slow down the degradation of cellulose and lignin (Jönsson & Martín, 2016).

In summary, *P. chrysogenum* demonstrated its adaptability, achieving optimal results with an 8‐week treatment for WS (cellulose reduction C.var = −30.17%, lignin reduction L.var = −34.59%, IVTD enhancement IVTD.var = 26.41%), while requiring 12 weeks for CW (C.var = −3.30%, L.var = −45.60%, IVTD.var = 18.6%) and a brief 4 weeks for OP (C.var = −4.3%, L.va = −3.22%, IVTD.var = 14.75%). *F*. sp. displayed noteworthy results after 8‐week treatment, particularly reducing cellulose content (C.var = −31.58% for WS) and improving IVTD (IVTD.var = 9.09% for WS). *F. oxysporum* excelled with an 8‐week treatment for CW (C.var = −2.75%, L.var = −42.56%, IVTD.var = 16.3%) and WS (C.var = −3.56%, L.var = −37.93%, IVTD.var = 13.4%), as well as 12 weeks for OP (C.var = 37.83%, L.var = −58.34%, IVTD.var = 58.5%). *F. solani* also achieved optimal results with an 8‐week treatment for OP (C.var = 25.94%, L.var = −55.53%, IVTD.var = 35.10%), CW (C.var = 6.26%, L.var = −43.72%, IVTD.var = 16.80%), and WS (C.var = 16.26%, L.var = −41.81%, IVTD.var = 51.31%). *P. crustosum* showed efficient cellulose degradation, with a 4‐week treatment for all substrates (e.g., CW C.var = −42.45%, L.var = 11.46%, IVTD.var = −3.85%). *C. viridescens* yielded optimal results for OP (C.var = −22.83%, L.var = −2.36%, IVTD.var = 10.08%) and WS (C.var = −18.64%, L.var = −6.69%, IVTD.var = 8.39%) in 4 weeks, while CW required a 12‐week treatment (C.var = 0.12%, L.var = −58.34%, IVTD.var = 18.45%). These findings provide valuable insights into the adaptability of these fungi to different substrates and optimal treatment durations, which is crucial for enhancing agricultural by‐product conversion processes and sustainability.

## 5 CONCLUSION

In conclusion, the current study highlights the multifaceted criteria for achieving optimal nutritional value through fungal treatment. The effectiveness of fungal treatment depends on the specific fungus used, the substrate used, and the duration of treatment. However, it is important to emphasize that the search for optimal nutritional value through fungal treatment is complex due to the dynamic interactions between different substrate components. Lignin degradation, cellulose modification, and variations in carbohydrate content have different effects on the overall nutritional value of treated substrates. For example, we can observe significant degradation of lignin but low digestibility, or vice versa.

*Fusarium oxysporum* and *F. solani* were the most selective lignin‐degrading fungi among the six tested, showing the greatest improvement in IVTD, NFC, and CP, and inducing significant chemical changes in wheat straw, olive pomace, and cedar wood. Among the substrates, wheat straw and olive pomace stood out as optimal choices with high nutritional value. The 8‐week treatment period proved to be optimal for achieving these nutritional improvements, striking a balance between effective lignin degradation and favorable changes in carbohydrate composition. These results provide valuable insights into the complexity of fungal treatment processes and offer practical guidance for optimizing the use of fungal treatment of lignocellulosic substrates in agricultural by‐products to enhance their nutritional value.

## CONFLICT OF INTEREST STATEMENT

The authors declare no conflict of interest.

## Data Availability

The data that support the findings of this study are available on request from the corresponding author.
